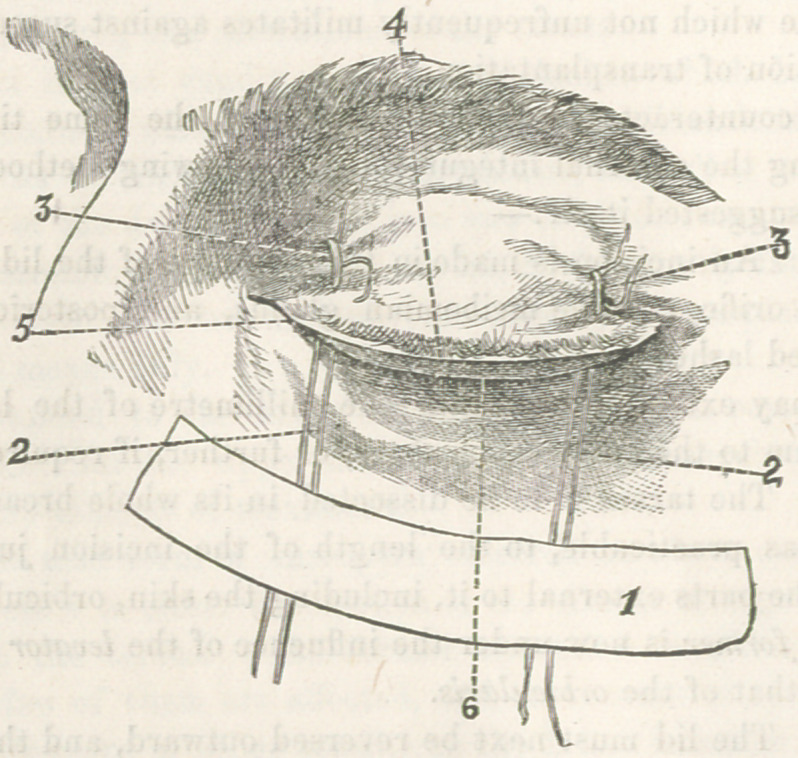# Entropion and Trichiasis of the Upper Lid; Their Radical Treatment, by an Operation, without Division of the Skin

**Published:** 1866-06

**Authors:** Joseph S. Hildreth

**Affiliations:** Late Brevet Lt.-Col. and Surgeon U.S. Vols., in charge of Desmarres (U.S. Army) Eye and Ear Hospital; Ophthalmic and Aural Surgeon to Cook County Hospital, Chicago, Ill.


ARTICLE XX.

### ENTROPION AND TRICHIASIS OF THE UPPER LID; THEIR RADICAL TREATMENT, BY AN OPERATION, WITHOUT DIVISION OF THE SKIN.

By JOSEPH S. HILDRETH, M.D., late Brevet Lt.-Col. and Surgeon U.S. Vols., in charge of Desmarres (U.S. Army) Eye and Ear Hospital; Ophthalmic and Aural Surgeon to Cook County Hospital, Chicago, Ill.

That abnormal condition of the lid which makes its free border turn inward is called Entropion. Its causes are various; but the operation here proposed is intended to apply, mainly, to that form of the affection resulting from atrophy, or loss of the mucous membrane, and alterations of the tarsus.

Trichiasis is that condition of the eyelashes which brings them into contact with the globe. Certain modifications are designated as districhiasis and tristrichiasis, according to the subdivision of the deviated lashes into two or three rows.

These distressing conditions of the lid, so frequently fatal to the eye involved, can be relieved, permanently, by surgical or mechanical means only.

When confined to the lower lid the procedure is comparatively simple; but the upper lid, from its peculiar mechanism, is much more difficult of treatment.

To relieve that form of entropion referred to above and trichiasis, I resort to either destruction of the cilia or change their position on the tarsus. The former operation is admissable when but few of them are affected, and on account of extreme age of the patient or other conditions the latter becomes inexpedient. With these exceptions, the operation of "transplantation" is always to be preferred. This consists in dissecting up that portion of the lid which contains the deviated cilia from the tarsus, and then causing it to become so attached to the cartilage that the lashes assume and remain in their proper position.

Before describing the manner in which this may be accomplished, a few words upon the mechanism of some of the parts involved will not be inappropriate.

1st. The position and functions of the orbicularis are such that, when contracting, it tends to draw the ciliary margin of the external integument of the lid *over* the free edge of its cartilage.

2d. The levator, when contracting, tends to draw the free edge of the cartilage *away* from the ciliary margin of the skin covering it.

Hence two antagonistic forces, *both* tending to produce a projection of the ciliary border of the skin covering the lid over the corresponding edge of the tarsus, and thereby deviating the cilia inward.

This constitutes the principle obstacle to be overcome, and the one which not unfrequently militates against success in the operation of transplantation.

To counteract this difficulty, and at the same time avoid dividing the external integument, the following method of operating suggested itself:—

1st. An incision is made in the free edge of the lid, anterior to the orifices of the meibomian glands, and posterior to the deviated lashes.

It may extend from within one millimetre of the lachrymal punctum to the outer commissure, or further, if required.

2d. The tarsus is to be dissected in its whole breadth, and, as far as practicable, to the length of the incision just made, from the parts external to it, including the skin, orbicularis, etc.

The *former* is now under the influence of the *levator* only, the *latter*, that of the *orbicularis*.

3d. The lid must next be reversed outward, and the tendon of the levator made to descend, so that a separate, coarse silk thread can be passed through it, close and parallel to the superior edge of the tarsus, and near both extremities of its separation from the external integuments. Both strands of each ligature must come through the mucous surface, and each loop should embrace horizontally about five millimetres of the tendon, taking care not to include any of the parts external to it.

The four strands of the two cords thus left projecting from the apperture of the lids should be left four or six inches long, in order to insure fastening to the cheek.

4th. Traction on these cords must next be made sufficient to bring the ciliary edge of the tarsus in contact with—in some cases below—the corresponding border of the opposite lid, and then firmly secured to the check by strips of adhesive plaster.

The skin and fibres of the orbicularis are now to be drawn upward and fixed to the tarsus in proper position by two *broad* stitches, inserted near the junction of the outer thirds with the inner third of the dissection of these parts from the tarsus.

The following cut, sketched immediately after an operation, shows the proper position of the parts:—

1, Represents method of securing cords which control the levator.

2, 2, The cords holding the levator.

3, 3, Stitches securing proper position of external integument.

4, Ciliary border.

5, Lachrymal punctum.

6, Border of tarsus, projecting, in this case, about two millimetres below the ciliary margin of the skin.

The strips of adhesive plaster should be numerous enough and the cords sufficiently long to guard against slipping.

The lower border is made to project a short distance below the ciliary margin of the external integuments, to allow for subsequent contraction. The extent to which it should project must depend upon the condition of its inner and lower edge. If this is well defined, it requires less than when rounded and irregular. In some cases, after adhesion has taken place, it may be well to remove a small portion of the lower border of the cartilage and thereby restore its normal shape.

The cords holding the levator should always be so arranged as not to rest on the cornea. They can be passed through the upper margin of the cartilage as well as the tendon, if the operator is fearful of their becoming detached too soon, and may remain in position a week, if desired; but three days, ordinarily, will be sufficient.

The outer stitches can be removed after the third day. But the removal of these as well as the long cords must of course depend upon the rapidity and firmness with which adhesion takes place.

Both eyes should be kept closed until the cords are removed.

Great care should be taken to so operate as to include *all* the deviated lashes. Should a few escape, they can be destroyed subsequently.

The advantages offered by this mode of operating are:—

1st. No wound of the external integument of the lid is required, which always produces more or less deformity.

2d. The tendency of the transplanted parts to suppurate, to fail to unite well, or of the cilia to subsequently fall out on account of imperfect nutrition, is avoided.

The circulation of the parts being but little interfered with, reunion is rapid and the result permanent.

The presence of the cords within the lids controlling the levator, like stitches required within the lids in other operations, does not prove to be a practical objection.

## Figures and Tables

**Figure f1:**